# Cardiac function and myocardial perfusion immediately following maximal treadmill exercise inside the MRI room

**DOI:** 10.1186/1532-429X-10-3

**Published:** 2008-01-15

**Authors:** Mihaela Jekic, Eric L Foster, Michelle R Ballinger, Subha V Raman, Orlando P Simonetti

**Affiliations:** 1 Dorothy M. Davis Heart and Lung Research Institute, 473 W 12^th ^Ave, Columbus, OH43210, USA; 2 Biomedical Engineering, The Ohio State University, 1080 Carmack Rd, Columbus, OH43210, USA; 3 Mechanical Engineering, The Ohio State University, 201 W 19^th ^Ave, Columbus, OH43210, USA; 4 Internal Medicine, The Ohio State University, 473 W 12^th ^Ave, Columbus, OH43210, USA; 5 Radiology, The Ohio State University, 1654 Upham Dr, Columbus, OH43210, USA

## Abstract

Treadmill exercise stress testing is an essential tool in the prevention, detection, and treatment of a broad spectrum of cardiovascular disease. After maximal exercise, cardiac images at peak stress are typically acquired using nuclear scintigraphy or echocardiography, both of which have inherent limitations. Although CMR offers superior image quality, the lack of MRI-compatible exercise and monitoring equipment has prevented the realization of treadmill exercise CMR.

It is critical to commence imaging as quickly as possible after exercise to capture exercise-induced cardiac wall motion abnormalities. We modified a commercial treadmill such that it could be safely positioned inside the MRI room to minimize the distance between the treadmill and the scan table. We optimized the treadmill exercise CMR protocol in 20 healthy volunteers and successfully imaged cardiac function and myocardial perfusion at peak stress, followed by viability imaging at rest. Imaging commenced an average of 30 seconds after maximal exercise. Real-time cine of seven slices with no breath-hold and no ECG-gating was completed within 45 seconds of exercise, immediately followed by stress perfusion imaging of three short-axis slices which showed an average time to peak enhancement within 57 seconds of exercise. We observed a 3.1-fold increase in cardiac output and a myocardial perfusion reserve index of 1.9, which agree with reported values for healthy subjects at peak stress. This study successfully demonstrates in-room treadmill exercise CMR in healthy volunteers, but confirmation of feasibility in patients with heart disease is still needed.

## Introduction

Since it was first proposed as a diagnostic tool for angina almost 75 years ago [1], treadmill exercise stress testing quickly became, and still remains, an essential tool in the detection and treatment of heart disease. The Bruce Treadmill Test, first published in 1963 [2], is the most commonly used exercise test protocol in the US [3,4], and has been shown to have high diagnostic and prognostic value [5,6]. The addition of imaging to the exercise stress test further improves sensitivity and specificity, providing greater diagnostic accuracy than exercise ECG alone [7,8]. According to some estimates, over 10 million stress studies are performed in the US each year in conjunction with nuclear or echocardiographic imaging [9]. While CMR stress imaging has demonstrated recent success and is potentially superior to stress echocardiography [10] and SPECT imaging [11], pharmacological stress has remained the only practical approach to CMR stress imaging due to the lack of MRI-compatible exercise and monitoring equipment. Exercise is preferred to pharmacologic stress testing because it links physical activity to symptoms and ischemia [12]. The exercise test itself offers additional important information such as exercise capacity, blood pressure response, development of arrhythmias, and the presence of symptoms such as chest pain during exercise [13]. Certain exercise parameters alone such as inability to complete 6 minutes of the Bruce treadmill protocol [14] and inability to reach 85% of age-predicted maximum heart-rate indicate significant risk of coronary events [15]. Pharmacologic stress is only indicated in those patients unable to undergo exercise stress testing for reasons such as de-conditioning, peripheral vascular disease, and orthopedic disabilities [16,17]. Stress CMR using a MRI-compatible bicycle ergometer has been previously demonstrated [18-22]), but fatigue of the quadriceps muscles in patients is a limitation to achieving target heart rate and maximal cardiovascular stress [23]. Untrained subjects will typically only achieve 80% – 90% of their treadmill maximum oxygen consumption on a bicycle ergometer [24].

While treadmill exercise is the physiologically preferred method of cardiovascular stress testing, it presents significant challenges for use with MRI. Treadmills are typically powered by electromagnetic motors and contain a multitude of ferromagnetic parts, precluding their use in close proximity to an MRI magnet. Bringing the treadmill, monitoring equipment, and staff into the MRI room would allow it to function much like a standard exercise stress lab. Furthermore, positioning the treadmill as close to the MRI patient table as possible would minimize the time from exercise to imaging. Any time delay is critical, since function imaging must be completed within 60 seconds post-exercise [12,25] to capture the exercise-induced wall motion abnormalities which may begin to disappear almost immediately after exercise [26-28]. Additionally, traveling any distance immediately following maximal exercise could be unsafe for severely ill or de-conditioned cardiac patients.

Besides the difficulties of safely operating exercise and monitoring equipment in the magnetic field of the MRI room, image acquisition during conditions of maximal cardiovascular stress is exceptionally challenging. Temporal resolution requirements are higher for exercise stress imaging due to elevated heart rates and rapid, heavy breathing. Total scan time must be minimized to acquire all data before exercise-induced ischemia is resolved. Breath-holding may be impossible for patients immediately following maximal exercise stress. Recent advances in MRI hardware and software, such as the TSENSE [29] method of dynamic parallel imaging, have resulted in significant improvements in the temporal and spatial resolution of real-time and single-shot imaging methods, significantly shortening scan time and eliminating the need for patient breath-hold.

The objectives of this study were to modify a treadmill for use inside the MRI room, to develop real-time imaging protocols for CMR of cardiac function and myocardial perfusion following maximal exercise, and to demonstrate the feasibility of in-room treadmill stress CMR in healthy volunteers. To our knowledge, real-time wall motion and perfusion imaging immediately after maximal treadmill exercise inside the MRI room has not been previously demonstrated.

## Methods

### Subjects

Twenty healthy subjects between the ages of 20 and 64(11 females, 9 males, mean age 39 ± 13 years, median age 44) underwent the exercise CMR protocol. The study protocol was approved by the Institutional Review Board at The Ohio State University. All participants gave written informed consent. The exclusion criteria were known or suspected CAD, inability to exercise, and standard contraindications to MRI.

### Equipment

#### MRI system and injector

We used a 1.5 Tesla MAGNETOM Avanto MRI system (Siemens Medical Solutions, Malvern, PA) with maximum gradient strength of 45 mT/m and slew rate of 200 mT/m/sec, 32-channel RF system, and a 12-channel array coil with 6 anterior and 6 posterior elements. The anterior surface coil contains a Hall-effect sensor that detects movement of the coil within the magnetic field. Gross motion of the coil detected by this sensor causes the MRI system to automatically perform a series of adjustments taking several seconds, or a failure to execute the next scan in the queue.

The MRI system is controlled via an MRI-compatible in-room console (Siemens Medical Solutions, Malvern, PA), and a start button located on the front panel of the magnet housing. The in-room console, designed primarily for interventional MRI applications, duplicates the functionality of the main imaging console.

A power injector (Spectris, Medrad Corp., Pittsburgh, PA) was outfitted with a manual control switch for operation from within the MRI room. The injection protocol was pre-programmed and loaded such that it could be executed immediately at the start of the perfusion scan from within the MRI room.

#### Treadmill modifications

**Figure 1:**
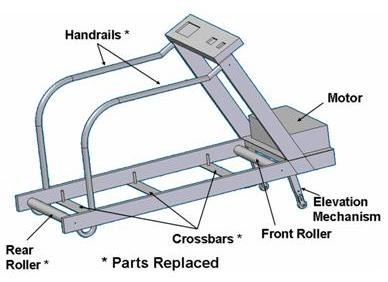
Treadmill modified by replacing ferromagnetic components to the rear of the motor with non-ferromagnetic stainless steel and aluminum.

**Figure 2:**
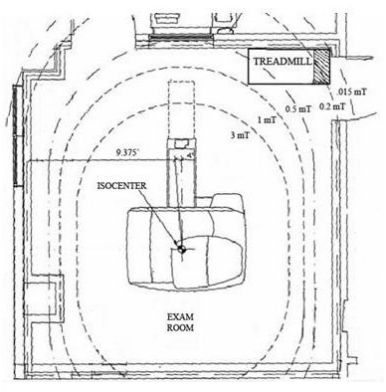
Magnetic field plot inside the MRI room showing the treadmill location during exercise testing. The remaining ferromagnetic treadmill components (shaded) are located in a field of only 0.2 mT (2 Gauss). A field of less than 5 Gauss is considered safe.

We modified a standard rehabilitation treadmill (Landis 8700, Randolph, NJ) by replacing the ferromagnetic components to the rear of the motor with non-ferromagnetic stainless steel and aluminum equivalents, as shown in Figure 1. Replacing these parts allowed us to safely position the treadmill in the corner of the MRI room such that the remaining ferromagnetic components, including the motor, front roller, and elevation mechanism, were located in a magnetic field of approximately 0.0002 Tesla (2 Gauss). A magnetic field of less than 5 Gauss poses little or no risk of attracting ferromagnetic objects [30]. Figure 2 shows the magnetic field plot, which was verified with a hand-held Gaussmeter, and the room layout for the MRI system. The outline of the treadmill positioned in the corner of the MRI room is shown, along with a shaded area indicating the location of the remaining ferromagnetic parts.

#### Patient monitoring

**Figure 3:**
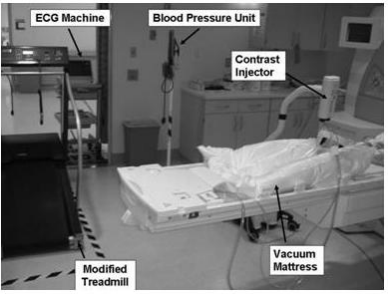
Experimental setup for the treadmill CMR test inside the MRI room. The ferromagnetic components, including the treadmill motor and the ECG system, are located in the corner of the MRI room where the magnetic field is less than 5 Gauss.

Continuous 12-Lead ECG monitoring of the patient is required during the exercise test [16]. To our knowledge there is currently no commercially available, MRI-compatible 12-lead stress ECG system. Therefore, we positioned a standard 12-lead ECG system at the entrance to the MRI room (Figure 3), close enough to monitor the subject both on the treadmill and on the MRI patient table when the patient is outside of the magnet bore. While inside the bore, the ECG is non-diagnostic due to magneto-hydrodynamic artifacts caused by blood flow within the magnetic field [31]. However, heart rate and rhythm can be monitored continuously with a wireless 3-electrode unit provided by the MRI manufacturer (Siemens Medical Solutions, Malvern, PA). This setup allowed us to quickly disconnect the patient from the 12-lead ECG system after exercise, while continuing to monitor heart rate with the 3-electrode unit. MRI-compatible manual and automatic non-invasive blood pressure equipment (Medrad, Inc., Pittsburgh, PA) was used to monitor blood pressure before, during, and after the stress test.

Thus, all equipment necessary to conduct the treadmill exercise test with continuous ECG and blood pressure monitoring, as well as the equipment necessary to control the MRI procedure, was positioned to allow the test to be performed within the MRI room. The stress testing team was able to remain in the room and in direct communication with the patient at all times.

#### Patient positioning

**Figure 4:**
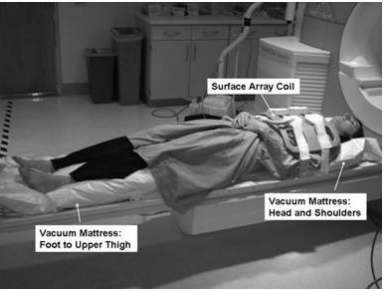
Two vacuum mattresses (head and shoulders, and foot to upper thigh) are used for repositioning the subject between rest and stress. Removal of air with a vacuum pump causes the mattresses to form a rigid mold around the subject.

Before exercise, the subject was positioned on the MRI table using two vacuum mattresses (Vac-Lok Cushions, MEDTEC, Orange City, IA), and slice localization and resting function scans were performed. One vacuum mattress was placed under the head and shoulders and the other under the legs extending from foot to upper thigh (Figure 4). Removal of air with a vacuum pump causes the mattresses to rigidly conform to the body. These devices are commonly used for repositioning of patients undergoing repeated radiation therapy sessions. This system was used to ensure that the subject returned to the same position after exercise such that stress imaging could be performed using the slice planes previously prescribed at rest.

### Pulse sequences

A real-time SSFP sequence with TR/TE of 2.3/1.0 msec and TSENSE acceleration factor of 3 was used for cine function imaging. Five slices were acquired in the short axis (SAX) direction, and one slice each in horizontal (HLA) and vertical (VLA) long axis directions. Temporal resolution of 57.8 ± 3.4 msec and spatial resolution of 2.9 mm × 3.7 mm × 8 mm were achieved with no breath-hold and no ECG gating. The seven slices were acquired in an average of 15 seconds.

0.1 mmol/kg gadolinium-DTPA was administered intravenously at a rate of 4 mL/s as a contrast agent for first-pass perfusion imaging. GRE-EPI with TR/TE of 5.8/1.2 msec and TSENSE acceleration rate of 2 was used to obtain 3 SAX slices each cardiac cycle. Saturation recovery time was 30 msec and the acquisition time per slice was 70 msec (96 × 160 matrix, 3.0 mm × 2.4 mm × 10 mm resolution).

### Exercise test protocol

The exercise test was performed by a nurse under the supervision of a cardiologist. One research team member performed the CMR scan, and another assisted in initial setup and started the stress scan within the MRI room.

**Figure 5:**
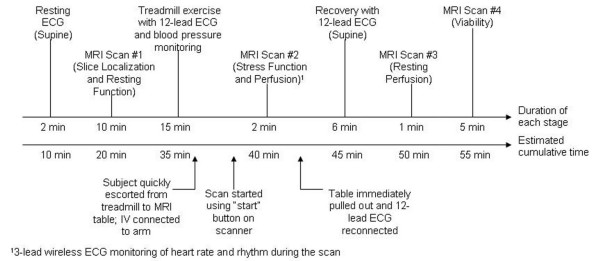
Timeline for the treadmill CMR test, including slice localization, rest and stress function, rest and stress perfusion, and viability. Both the duration of each stage of the test and the estimated cumulative time are shown.

The exercise CMR protocol is summarized in Figure 5. Patient preparation included insertion of an intravenous (IV) needle and the standard placement of both the 12-lead and the 3-electrode wireless ECG electrodes on the chest [16]. Supine 12-lead ECG and blood pressure (BP) were recorded at rest. The supine resting ECG was required for direct comparison with the supine recovery ECG post-exercise. Next, subjects were positioned on the MRI table using the vacuum mattresses. Air was removed from the mattresses through a vacuum line located inside the MRI room.

Slice localization by single-shot SSFP was followed by resting cine imaging. The cine function sequence was set up to scan each slice position for 1.8 seconds, while the temporal resolution varied depending on the size of the patient and the resulting field of view. A test acquisition for first-pass perfusion was performed without contrast agent. The pulse sequences were queued up for stress imaging such that they could be executed automatically from the scan start button located on the magnet. The subject was then removed from the magnet, with care taken not to pull the table all the way out of the magnet, and not to move the surface array coil too drastically to avoid activation of the Hall-effect sensor. Either of these actions was found to cause the system to repeat adjustments prior to the start of the stress scan, causing a delay and/or failure to execute the function scan.

Next, the subjects exercised on a treadmill positioned inside the MRI room, as shown in Figure 3. The treadmill speed and elevation were progressively increased every three minutes following the standard Bruce protocol. 12-lead ECG was continuously monitored during exercise. Blood pressure was measured and a hard copy of the ECG was obtained at the midpoint of each Bruce protocol stage. As with conventional stress testing, subjects were continuously monitored by a nurse and/or physician who could stop the test at any time based on recognition of adverse endpoints or in response to the subject's request. After reaching their exercise limit or the maximum predicted heart rate (MPHR) based on age (220-age), the 12-lead ECG was disconnected and the subjects were quickly escorted to the MRI table. Heart rate and rhythm monitoring continued with the 3-electrode wireless unit. The surface coil was placed on the chest, the contrast injector was connected to the previously inserted IV in the subject's arm, and the MRI table was returned to the original position inside the magnet. The previously prepared cine and first-pass perfusion scans were started using the start button located on the magnet; stress function was executed first, followed by stress perfusion. The time from end of exercise to start of imaging (Tstart) was recorded. A member of the team started the injection protocol as soon as the audible change from the cine pulse sequence to the first-pass pulse sequence was detected. The subject remained inside the magnet bore for approximately 90 seconds for stress imaging.

Immediately following imaging, the MRI table was pulled out and 12-lead ECG and blood pressure were recorded for 6–8 minutes during supine recovery on the table. Following recovery, the subject was again moved into the magnet bore, and resting perfusion and late Gadolinium enhancement (LGE) viability images were obtained. We followed the acquisition order described by Klem et al. [9], which included stress perfusion followed by rest perfusion and finally viability.

### Data analysis

Argus (Siemens Medical Solutions, Inc., Malvern, PA) was used to compute the ratio of cardiac output (CO) at stress and rest by visually selecting the end diastolic and end systolic frames, and manually tracing the endocardial borders. The reduction in scan time using the real-time TSENSE algorithm enabled us to acquire 5 SAX slices. Therefore, we were able to select the 3 slices that were most closely aligned between rest and stress and thus to compensate for any repositioning error.

The myocardial perfusion reserve index (MPRI) was computed for one mid-ventricular slice using Argus. For these healthy volunteers, uniform myocardial perfusion was assumed in the analysis. The slope of the myocardial signal intensity curve was computed for the entire slice and normalized by the slope of the blood pool signal intensity curve [32]. The stress slope was divided by the rest slope to obtain a global estimate of MPRI.

## Results

The entire procedure, from initial setup to completion of the LGE scan, took an average of one hour. The results are displayed in Table 1 for the 19 out of 20 subjects for whom at least one of the stress scans (function or perfusion) was successful. Two perfusion scans were unsuccessful due to IV failure, and two function scans failed to start due to activation of the Hall-effect coil-motion sensor when the coil was moved too drastically inside the magnetic field. The first time the function scan failed, no usable data was obtained, whereas in the second case perfusion data was acquired. The former case is not presented in Table 1 since no image data was obtained.

**Table 1:** Results of the treadmill CMR test in 19 healthy subjects. The heart rate at peak exercise and start of imaging is expressed as %MPHR. The times from end of exercise to start of imaging, from end of exercise to end of function imaging, and from end of exercise to peak myocardial enhancement are shown.

**Subject**	**Age**	**Bruce Stage**	**Max HR (%MPHR)**	**Start Img HR (%MPHR)**	**StressCO/Rest CO**	**MPRI**	**Tstart (sec)**	**Tend_fcn (sec)**	**Tpeak_enhanc (sec)**
1	20	5	92	73	3.5	2.1	25	40	47
2	35	6	92	69	3.5	1.8	27	43	59
3	35	4	102	98	2.5	1.8	25	39	48
4	25	5	102	95	3.1	2.1	24	38	50
5	25	5	87	65	3.8	1.4	37	51	63
6	25	5	85	63	3.5	2.0	33	47	56
7	20	5	93	85	3.3	N/A	27	42	N/A
8	35	5	96	82	3.2	1.8	29	44	58
9	31	5	96	77	3.5	1.4	27	42	57
10	55	5	99	83	2.3	1.7	36	52	60
11	46	4	101	83	3.6	3.5	32	48	61
12	58	4	102	81	3.1	2.0	31	46	56
13	44	5	98	82	3.1	N/A	32	48	N/A
14	50	5	100	N/A	N/A	1.4	32	N/A	N/A
15	44	3	101	87	2.1	1.5	30	43	57
16	46	5	109	99	3.1	1.8	29	44	59
17	48	4	99	92	2.0	2.1	32	45	61
18	64	3	113	101	2.5	1.3	34	49	65
19	44	5	100	30	3.8	2.2	28	43	59
**Mean**	**13**	**5**	**98**	**84**	**3.1**	**1.9**	**30**	**45**	**57**
**SD**	**13**	**1**	**7**	**11**	**0.6**	**0.5**	**4**	**4**	**5**

HR: heart rate, MPHR: maximum predicted heart rate based on age, CO: cardiac output, MPRI: myocardial perfusion reserve index

**Figure 6:**
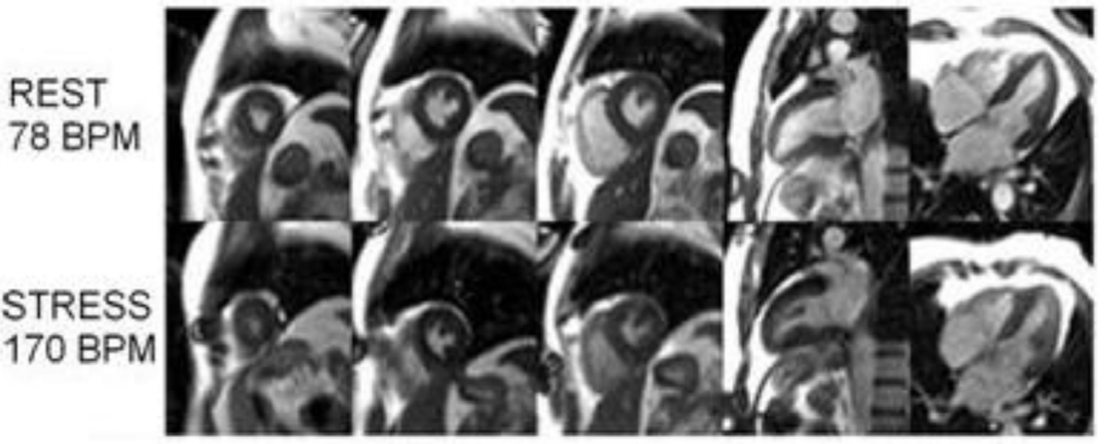
End-systolic frames clearly depicting increased contractility from rest (top) to stress (bottom). From left to right, three SAX, one VLA, and one HLA views are shown.

**Figure 7:**
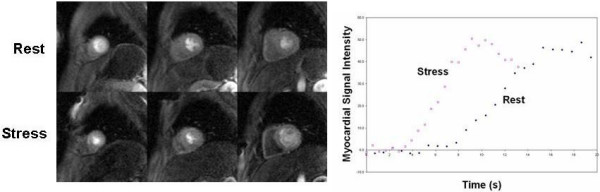
Three short-axis first-pass perfusion images at rest and during exercise stress are shown on the left. Graph on the right shows myocardial signal intensity in a mid-ventricular slice during the first pass of the contrast agent, clearly depicting the increased slope from rest to stress.

Heart rate at peak exercise and at start of imaging is expressed as percent of maximum predicted heart rate based on age (MPHR = 220-age). Cardiac output increased by a factor of 3.1 ± 0.6 from rest to stress. Increased contractility is clearly depicted in the end-systolic images at rest and stress displayed in Figure 6. Figure 7 shows representative first-pass perfusion images at stress and rest, and the myocardial signal intensity curves measured during the first pass of the contrast agent through the heart. The increase in slope from rest to stress due to increased flow through the myocardium is clearly depicted, which supports quantitative perfusion analysis that yielded an average MPRI of 1.9 ± 0.5. The scan commenced an average of 30 ± 4 seconds after exercise while function imaging was completed an average of 45 ± 4 seconds post-exercise. The time from end of exercise to peak myocardial enhancement in the perfusion images was 57 ± 5 seconds. Image quality was sufficient for visual assessment of wall motion in all left ventricular segments, and no subject showed any evidence of exercise-induced wall motion or perfusion abnormality.

## Discussion

We have successfully demonstrated the ability to acquire stress cardiac function and myocardial perfusion images immediately following maximal exercise on a treadmill inside the MRI room. Although two clinical and two research staff members were involved in the experiment, we have since streamlined the process to include only a nurse, a supervising cardiologist, and a technologist performing the scan. In the two cases of accidental Hall-effect sensor activation due to significant coil movement within the magnetic field, several seconds of automatic system adjustments were triggered and the cine sequence failed to start. The first time this occurred, contrast was not injected and no usable image data obtained. The second time, we injected contrast at the start of the perfusion scan, enabling us to quantify MPRI in the absence of function data (Subject #14 in Table 1). Once we recognized the cause of this problem, we were able to avoid it in subsequent studies.

The increase in cardiac output by a factor of 3.1 was comparable to a previous study in healthy young sedentary subjects undergoing maximal treadmill exercise, which showed a factor of 3.2 cardiac output increase in men and 2.9 in women [33]. In addition, the MPRI of 1.9 ± 0.5 fell within the normal range of MPRI (2.3 ± 1.3, averaged over six segments) previously found with adenosine CMR in patients with negative coronary angiograms [32]. No subject demonstrated chronotropic incompetence, i.e. the inability to reach 85% of MPHR based on age. Except for the oldest subject (age 64), all subjects reached at least stage 4 of the Bruce Protocol (exercise time >9 minutes). The spatial resolution of 2.9 × 3.7 mm was adequate for wall motion assessment and the delineation of endocardial borders for calculating functional parameters.

Rerkpattanapipat et al. [34] have shown the feasibility of detecting coronary artery stenoses by exercise CMR using a treadmill positioned outside the MRI room, requiring the subject to walk about 20 feet from the treadmill to the MRI system. With the use of segmented cine pulse sequences requiring a 4 to 8 second breath-hold, the sensitivity and specificity to detect >70% coronary artery diameter narrowing in 27 patients were 79% and 85%, respectively, based on wall motion alone. The application of real-time imaging for wall motion assessment is an advantage of the current work over that published by Rerkpattanapipat. Real-time imaging methods eliminate the requirements of breath-holding and ECG gating, and provide inherently faster scan time allowing the acquisition of five short-axis and two long-axis views. This enabled sufficient coverage for the assessment of wall motion abnormalities using the standard 17-segment model of the left ventricle [35]. The temporal resolution of the real-time TSENSE acquisition in our study was limited to 57.8 milliseconds, which may be insufficient to accurately resolve wall motion abnormalities at high heart rates. A 32-channel surface array coil may permit the use of higher SENSE acceleration rates and improved temporal resolution [36]. An additional advancement in our protocol was the addition of stress and rest perfusion and LGE viability information. Klem et al. [9] have shown that the combination of rest perfusion and LGE is valuable in distinguishing true perfusion defects from artifacts, and improves the diagnostic accuracy over stress perfusion alone.

While ECG triggering was not required for real-time cine, it was required for perfusion imaging. Triggering difficulties were encountered in some cases due to heavy respiratory chest wall motion post-exercise, but did not prevent the interpretation of the first-pass image data. A mistimed trigger affects the phase of the cardiac cycle at which the image is acquired, but not image quality since we are using a single-shot sequence. Improved R-wave detection algorithms and triggering devices are being explored to address this problem, and preliminary experience indicates improved triggering performance. The next phase of the project is to test whether this combined approach of exercise stress with CMR of function, perfusion, and viability will provide improved diagnostic accuracy for CAD over existing methods.

The work by Rerkpattanapipat et al.[34], provided a critical step toward developing a feasible clinical treadmill exercise CMR system. We sought to extend this work by moving the treadmill into the MRI room, which achieves several goals. First, it minimizes any time delay between the end of exercise and imaging; as with stress echocardiography, this delay must be minimized in order for post-stress imaging to adequately capture any stress-induced wall motion or perfusion abnormalities. Second, there is variability across MR centers in the space available outside the MRI room for treadmill, monitoring equipment, and staff required to execute the test; we felt that moving the treadmill into the MRI room would help insure patient safety and privacy across this variability. While our experiments were performed safely under carefully controlled conditions, there was still a risk of accident due to the ferromagnetic treadmill motor. We are currently developing a totally magnet-compatible treadmill that can be safely placed immediately adjacent to the MRI patient table. This will minimize the time delay between exercise and imaging, and provide a safe means of CMR exercise stress testing at any MRI field strength. A totally MRI-compatible treadmill will enable a CMR magnet room be configured similar to a stress echocardiography lab, with the examination bed positioned immediately behind or adjacent to the treadmill. Such a configuration will enable patients to get off of the treadmill and directly on to the scanner table, minimizing both the delay in commencing imaging (particularly important in cardiac patients who are expected to be less mobile than healthy subjects) and patient risk by eliminating any travel immediately after peak exercise.

Besides the detection of CAD, exercise CMR can potentially create new avenues for research and clinical practice, such as stress evaluation of right ventricle (RV) dysfunction. One of the limitations of echocardiography is the assessment of RV function due to complex RV shape and orientation combined with poor endocardial definition [37-39]. In the presence of lung disease or chronic obstructive pulmonary disease (COPD), frequently associated with pulmonary hypertension, poor acoustic windows may further limit echocardiography. Our group has previously found that multi-detector row computed tomography is comparable to CMR in quantifying RV volumes and ejection fraction in patients with tetralogy of Fallot and transposition of the great arteries [40], but computed tomography is limited by radiation exposure and insufficient temporal resolution to image the rapid heart rates associated with exercise stress. This is just one example of many potential applications for exercise stress CMR.

## Conclusion

Despite tremendous advances in cardiovascular testing in the last few decades, consistently accurate stress imaging remains an important target for technology development to reduce uncertainty in the diagnosis and treatment of patients with many forms of cardiovascular disease. CMR already provides, in a single examination, high resolution assessment of stress wall motion, stress perfusion, and myocardial viability. This study has successfully demonstrated in-room treadmill exercise CMR in healthy volunteers, but confirmation of feasibility in patients with heart disease is still needed. If successful, the combination of exercise stress testing and CMR may have a significant impact on the clinical diagnosis and treatment of cardiovascular disease.

## Competing interests

We are currently applying for a patent relating to the content of this manuscript.

## References

[B1] Goldhammer S, Scherf D (1932). Elektrokardiographische unterschungen bei kranken mit angina pectoris ("ambulatorischer Typus"). Ztschr f klin Med.

[B2] Bruce RA, Blackmon JR, Jones JW, Strait G (1963). Exercising Testing in Adult Normal Subjects and Cardiac Patients. Pediatrics.

[B3] Myers J, Voodi L, Umann T, Froelicher VF (2000). A survey of exercise testing: methods, utilization, interpretation, and safety in the VAHCS. J Cardiopulm Rehabil.

[B4] Lear SA, Brozic A, Myers JN, Ignaszewski A (1999). Exercise stress testing. An overview of current guidelines. Sports Med.

[B5] Ashley EA, Myers J, Froelicher V (2000). Exercise testing in clinical medicine. Lancet.

[B6] Tavel ME, Shaar C (1999). Relation between the electrocardiographic stress test and degree and location of myocardial ischemia. Am J Cardiol.

[B7] Lee TH, Boucher CA (2001). Clinical practice. Noninvasive tests in patients with stable coronary artery disease. N Engl J Med.

[B8] Garber AM, Solomon NA (1999). Cost-effectiveness of alternative test strategies for the diagnosis of coronary artery disease. Ann Intern Med.

[B9] Klem I, Heitner JF, Shah DJ, Sketch MH, Behar V, Weinsaft J, Cawley P, Parker M, Elliott M, Judd RM, Kim RJ (2006). Improved detection of coronary artery disease by stress perfusion cardiovascular magnetic resonance with the use of delayed enhancement infarction imaging. J Am Coll Cardiol.

[B10] Nagel E, Lehmkuhl HB, Bocksch W, Klein C, Vogel U, Frantz E, Ellmer A, Dreysse S, Fleck E (1999). Noninvasive Diagnosis of Ischemia-Induced Wall Motion Abnormalities With the Use of High-Dose Dobutamine Stress MRI: Comparison With Dobutamine Stress Echocardiography. Circulation.

[B11] Schwitter J (2005). MR-IMPACT: Magnetic Resonance Imaging for Myocardial Perfusion Assessment in Coronary Artery Disease Trial. European Society of Cardiology Congress.

[B12] Armstrong WF, Zoghbi WA (2005). Stress echocardiography: current methodology and clinical applications. J Am Coll Cardiol.

[B13] Tavel ME (2001). Stress Testing in Cardiac Evaluation: Current Concepts With Emphasis on the ECG 10.1378/chest.119.3.907. Chest.

[B14] Bruce RA, Hornsten TR (1969). Exercise stress testing in evaluation of patients with ischemic heart disease. Prog Cardiovasc Dis.

[B15] Roger VL, Jacobsen SJ, Pellikka PA, Miller TD, Bailey KR, Gersh BJ (1998). Prognostic value of treadmill exercise testing: a population-based study in Olmsted County, Minnesota. Circulation.

[B16] Fletcher GF, Balady GJ, Amsterdam EA, Chaitman B, Eckel R, Fleg J, Froelicher VF, Leon AS, Piña IL, Rodney R, Simons-Morton DA, Williams MA, Bazzarre T (2001). Exercise standards for testing and training: a statement for healthcare professionals from the American Heart Association. Circulation.

[B17] Marwick TH (2003). Stress echocardiography. Heart.

[B18] Cheng CP, Herfkens RJ, Taylor CA (2003). Inferior vena caval hemodynamics quantified in vivo at rest and during cycling exercise using magnetic resonance imaging. Am J Physiol Heart Circ Physiol.

[B19] Cheng CP, Herfkens RJ, Taylor CA (2003). Abdominal aortic hemodynamic conditions in healthy subjects aged 50–70 at rest and during lower limb exercise: in vivo quantification using MRI. Atherosclerosis.

[B20] Taylor CA, Cheng CP, Espinosa LA, Tang BT, Parker D, Herfkens RJ (2002). In vivo quantification of blood flow and wall shear stress in the human abdominal aorta during lower limb exercise. Ann Biomed Eng.

[B21] Niezen RA, Doornbos J, van der Wall EE, de Roos A (1998). Measurement of aortic and pulmonary flow with MRI at rest and during physical exercise. J Comput Assist Tomogr.

[B22] Hjortdal VE, Emmertsen K, Stenbøg E, Fründ T, Schmidt MR, Kromann O, Sørensen K, Pedersen EM (2003). Effects of exercise and respiration on blood flow in total cavopulmonary connection: a real-time magnetic resonance flow study. Circulation.

[B23] Gibbons RJ, Balady GJ, Bricker JT, Chaitman BR, Fletcher GF, Froelicher VF, Mark DB, McCallister BD, Mooss AN, O'Reilly MG, Winters WL, Gibbons RJ, Antman EM, Alpert JS, Faxon DP, Fuster V, Gregoratos G, Hiratzka LF, Jacobs AK, Russell RO, Smith SC (2002). ACC/AHA 2002 guideline update for exercise testing: summary article. A report of the American College of Cardiology/American Heart Association Task Force on Practice Guidelines (Committee to Update the 1997 Exercise Testing Guidelines). J Am Coll Cardiol.

[B24] Miyamura M, Honda Y (1972). Oxygen intake and cardiac output during treadmill and bicycle exercise. J Appl Physiol.

[B25] Armstrong WF, Pellikka PA, Ryan T, Crouse L, Zoghbi WA (1998). Stress echocardiography: recommendations for performance and interpretation of stress echocardiography. Stress Echocardiography Task Force of the Nomenclature and Standards Committee of the American Society of Echocardiography. J Am Soc Echocardiogr.

[B26] Salustri A, Pozzoli MM, Hermans W, Ilmer B, Cornel JH, Reijs AE, Roelandt JR, Fioretti PM (1992). Relationship between exercise echocardiography and perfusion single-photon emission computed tomography in patients with single-vessel coronary artery disease. Am Heart J.

[B27] Iliceto S, D'Ambrosio G, Sorino M, Papa A, Amico A, Ricci A, Rizzon P (1986). Comparison of postexercise and transesophageal atrial pacing two-dimensional echocardiography for detection of coronary artery disease. Am J Cardiol.

[B28] Dymond DS, Foster C, Grenier RP, Carpenter J, Schmidt DH (1984). Peak exercise and immediate postexercise imaging for the detection of left ventricular functional abnormalities in coronary artery disease. Am J Cardiol.

[B29] Kellman P, Epstein FH, McVeigh ER (2001). Adaptive sensitivity encoding incorporating temporal filtering (TSENSE). Magn Reson Med.

[B30] Kettenbach J, Kacher DF, Kanan AR, Rostenberg B, Fairhurst J, Stadler A, Kienreich K, Jolesz FA (2006). Intraoperative and interventional MRI: recommendations for a safe environment. Minim Invasive Ther Allied Technol.

[B31] Fischer SE, Wickline SA, Lorenz CH (1999). Novel real-time R-wave detection algorithm based on the vectorcardiogram for accurate gated magnetic resonance acquisitions. Magn Reson Med.

[B32] Nagel E, Klein C, Paetsch I, Hettwer S, Schnackenburg B, Wegscheider K, Fleck E (2003). Magnetic resonance perfusion measurements for the noninvasive detection of coronary artery disease. Circulation.

[B33] Ogawa T, Spina RJ, Martin WH, Kohrt WM, Schechtman KB, Holloszy JO, Ehsani AA (1992). Effects of aging, sex, and physical training on cardiovascular responses to exercise. Circulation.

[B34] Rerkpattanapipat P, Gandhi SK, Darty SN, Williams RT, Davis AD, Mazur W, Clark HP, Little WC, Link KM, Hamilton CA, Hundley WG (2003). Feasibility to detect severe coronary artery stenoses with upright treadmill exercise magnetic resonance imaging. Am J Cardiol.

[B35] Cerqueira MD, Weissman NJ, Dilsizian V, Jacobs AK, Kaul S, Laskey WK, Pennell DJ, Rumberger JA, Ryan T, Verani MS, American Heart Association Writing Group on Myocardial Segmentation and Registration for Cardiac Imaging (2002). Standardized myocardial segmentation and nomenclature for tomographic imaging of the heart: a statement for healthcare professionals from the Cardiac Imaging Committee of the Council on Clinical Cardiology of the American Heart Association. Circulation.

[B36] Wintersperger BJ, Reeder SB, Nikolaou K, Dietrich O, Huber A, Greiser A, Lanz T, Reiser MF, Schoenberg SO (2006). Cardiac CINE MR imaging with a 32-channel cardiac coil and parallel imaging: impact of acceleration factors on image quality and volumetric accuracy. J Magn Reson Imaging.

[B37] Alam M, Hedman A, Nordlander R, Samad B (2003). Right ventricular function before and after an uncomplicated coronary artery bypass graft as assessed by pulsed wave Doppler tissue imaging of the tricuspid annulus. Am Heart J.

[B38] David JS, Tousignant CP, Bowry R (2006). Tricuspid annular velocity in patients undergoing cardiac operation using transesophageal echocardiography. J Am Soc Echocardiogr.

[B39] Marcu CB, Beek AM, Van Rossum AC (2006). Cardiovascular magnetic resonance imaging for the assessment of right heart involvement in cardiac and pulmonary disease. Heart Lung Circ.

[B40] Raman SV, Cook SC, McCarthy B, Ferketich AK (2005). Usefulness of multidetector row computed tomography to quantify right ventricular size and function in adults with either tetralogy of Fallot or transposition of the great arteries. Am J Cardiol.

